# Stomatal Sensitivity to Vapor Pressure Deficit and the Loss of Hydraulic Conductivity Are Coordinated in *Populus euphratica*, a Desert Phreatophyte Species

**DOI:** 10.3389/fpls.2020.01248

**Published:** 2020-08-14

**Authors:** Da-Yong Fan, Qing-Lai Dang, Cheng-Yang Xu, Chuang-Dao Jiang, Wang-Feng Zhang, Xin-Wu Xu, Xiao-Fang Yang, Shou-Ren Zhang

**Affiliations:** ^1^College of Forestry, Beijing Forestry University, Beijing, China; ^2^Faculty of Natural Resources Management, Lakehead University, Thunder Bay, ON, Canada; ^3^State Key Laboratory of Vegetation and Environmental Change, Institute of Botany, The Chinese Academy of Sciences, Beijing, China; ^4^The Key Laboratory of Oasis Eco-agriculture, Xinjiang Production and Construction Corps, Shihezi University, Shihezi, China; ^5^China Meteorological Administration, Beijing, China

**Keywords:** hydraulic model, xylem cavitation, leaf specific hydraulic conductance, stomatal conductance, water homeostasis

## Abstract

There are considerable variations in the percentage loss of hydraulic conductivity (*PLC*) at mid-day minimum water potential among and within species, but the underpinning mechanism(s) are poorly understood. This study tested the hypothesis that plants can regulate leaf specific hydraulic conductance (*K*_l_) *via* precise control over *PLC* under variable Δ*Ψ* (water potential differential between soil and leaf) conditions to maintain the *−m/b* constant (*−m*: the sensitivity of stomatal conductance to *VPD*; *b*: reference stomatal conductance at 1.0 kPa *VPD*), where *VPD* is vapor pressure deficit. We used *Populus euphratica*, a phreatophyte species distributed in the desert of Northwestern China, to test the hypothesis. Field measurements of *VPD*, stomatal conductance (*g*_s_), *g*_s_ responses to *VPD*, mid-day minimum leaf water potential (*Ψ*_lmin_), and branch hydraulic architecture were taken in late June at four sites along the downstream of Tarim River at the north edge of the Taklamakan desert. We have found that: 1) the *−m/b* ratio was almost constant (=0.6) across all the sites; 2) the average *Ψ*_50_ (the xylem water potential with 50% loss of hydraulic conductivity) was *−*1.63 MPa, and mid-day *PLC* ranged from 62 to 83%; 3) there were tight correlations between *Ψ*_50_ and wood density/leaf specific hydraulic conductivity (*k*_l_) and between specific hydraulic conductance sensitivity to water potential [d(*k*_s_)/dln(*−Ψ*)] and specific hydraulic conductivity (*k*_s_). A modified hydraulic model was applied to investigate the relationship between *g*_s_ and *VPD* under variable Δ*Ψ* and *K*_l_ conditions. It was concluded that *P. euphratica* was able to control *PLC* in order to maintain a relatively constant *−m/b* under different site conditions. This study demonstrated that branchlet hydraulic architecture and stomatal response to *VPD* were well coordinated in order to maintain relatively water homeostasis of *P. euphratica* in the desert. Model simulations could explain the wide variations of *PLC* across and within woody species that are often observed in the field.

## Introduction

A global convergence has been demonstrated in the relationship between drought-induced embolism and daily minimum xylem water potential (Choat et al., 2012; Choat et al., 2018). The safety margin of the plant hydrautic system refers to the difference between the daily minimum xylem water potential and the xylem water potential at which 50% of the hydraulic conductance is lost due to the cavitation of xylem vessels. Plants are generally able to maintain the integrity of their hydraulic system within the safety margin by the stomatal regulation of water loss to maximize the carbon gain without the risk of catastrophic hydraulic failure. However, the functional association between minimum xylem water potential and hydraulic safety does not prove that all the plants can control embolisms to the same extent because *PLC* is a function of water potential, *Ψ*_50_, and the slope of the cavitation vulnerability curve. As such, there are considerable inter- and intra-specific variations in *PLC* at the daily minimum xylem water potential (Pockman and Sperry, 2000; Johnson et al., 2009b; Fan et al., 2018). However, the underpinning mechanism(s) are not fully understood.

The stomatal regulation of xylem pressure is a function of vapor pressure deficit (*VPD*), leaf specific hydraulic conductance (*K*_l_), soil water potential (*Ψ*_s_), and leaf water potential (*Ψ*_l_) (see **Table 1** for the definitions of major acronyms/symbols in the present study) according to the following simplified hydraulic model (Oren et al., 1999; Landsberg et al., 2017):

(1) $$g_{l}=K_{l}\cdot \left(1/VPD\right)\cdot \left(\Psi_{s}-\Psi_{l}\right)$$

**Table 1:** List of symbols, abbreviations and their units.

Symbol/Abbreviations	Definition	Units
*a*	vulnerability curve steepness	
*b*	reference conductance at *VPD* = 1 kPa	mmol m^−2^ s^−1^
d(*k*_s_)/dln(−*Ψ*)	sensitivity of *k*_s_ to decreasing water potential	kg m^−1^ MPa^−2^ s^−1^
*g*_bl_	the boundary layer conductance to water vapor	mmol m^−2^ s^−1^
*g*_l_	leaf conductance to water vapor	mmol m^−2^ s^−1^
*g*_s_	stomatal conductance	mmol m^−2^ s^−1^
*g*_sm_	the maximum physiological *g*_s_	mmol m^−2^ s^−1^
Huber value	the total cross-section sapwood area per unit leaf area	m^2^ m^−2^
*k*_l_	leaf specific hydraulic conductivity,	kg m^−1^ MPa^−1^ s^−1^
*k*_s_	specific conductivity	kg m^−1^ MPa^−1^ s^−1^
*K*_h_	the maximum hydraulic conductivity	kg m MPa^−1^ s^−1^
*K*_hi_	the hydraulic conductivity measured at pressure i	kg m MPa^−1^ s^−1^
*K*_l_	leaf specific hydraulic conductance	mmol m^−2^ MPa^−1^ s^−1^
−*m*	the sensitivity of *g_s_* to *VPD*	mmol m^−2^ s^−1^ ln (kPa)^-1^
*−m/b*	the sensitivity of *g_s_* to *VPD* standardized by the stomatal conductance at 1.0 kPa *VPD*	
*PLC*	percentage loss of hydraulic conductivity	
*T*_r_	transpiration rate	mol m^−2^ s^−1^
*VPD*	leaf vapor pressure deficit	kPa
*WD*	woody density	g dry mass cm^−3^
*Ψ*	the negative of the injection pressure for vulnerability curve establishment	MPa
*Ψ*_l_	leaf water potential	MPa
*Ψ*_min_	daily minimum branchlet xylem water potential	MPa
*Ψ*_lmin_	daily minimum leaf xylem water potential	MPa
*Ψ*_s_	soil water potential	MPa
Δ*Ψ*	water potential differential between soil and leaf	MPa

Where *g*_l_ is leaf conductance to water vapor, which is a function of boundary layer conductance to water vapor (*g*_bl_) and stomatal conductance (*g*_s_). It has been demonstrated that the sensitivity of stomatal conductance to *VPD* (−*m*) has a close relationship with the stomatal conductance at 1 kPa VPD (*b*) and the −*m/b* ratio is found to be 0.6 for various mesic species across a variety of growth forms and habitats (Dang et al., 1997; Oren et al., 1999; Bucci et al., 2005; Landsberg et al., 2017) and the relationship is described as follows:

(2) $$g_{s}=m\cdot \ln VPD+b$$

The above models predict that if *K*_l_ decreases due to xylem cavitation, the −*m/b* ratio will need to increase because a more sensitive stomatal response is required to keep transpiration and Δ*Ψ* (=*Ψ_S_ – Ψ_l_*, *i.e.*, water potential difference between soil and leaf) relatively constant (Oren et al., 1999; Landsberg et al., 2017). Furthermore, the large variations in *PLC* (which regulates *K*_l_) will likely lead to variations in the −*m/b* ratio. However, it is unlikely in nature that Δ*Ψ* remains constant when *K*_l_ varies even in isohydric species (Mcdowell, 2011). Therefore, the assumption of constant Δ*Ψ* needs to be relaxed. In this study, we simultaneously examined stomatal conductance to water vapor pressure deficit, the response of branch and leaf hydraulic conductance to water potential differential between leaf and soil, and xylem vulnerability to cavitation. We conducted the study on four populations of a desert phreatophyte tree species, *Populus euphratica*, along a gradient of water table depths. We test the hypothesis that if stomata are perfectly efficient in regulating leaf water status as indicated by a constant −*m*/*b* both within and between species (Oren et al., 1999; Ewers et al., 2000), plants would fine-tune *K*_l_ *via* precise control over *PLC* (control the embolism degree) under variable Δ*Ψ* and *K*_l_ conditions. The results can provide an explanation for the considerable inter- and intra-specific variations in *PLC* at the daily minimum xylem water potential in the field.

From Equation 1, *g*_l_ can be obtained with the input of *K*_l_, *VPD*, *Ψ*_l_, and *Ψ*_s_, while *K*_l_ is the product of maximum *K*_l_ and *PLC*. *g*_s_ can be obtained provided *g*_bl_ is known. Then −*m*/*b* can be calculated based on Equation 2. Among all parameters required by the model, *K*_l_, *VPD*, *Ψ*_l_, and *PLC* can be measured/calculated in the field. In desert environment, *g*_bl_ is large and has minor impact on the model simulation (Comstock and Mencuccini, 1998; Oren et al., 1999). As such, the only obstacle to verify the above hypothesis for trees with a deep root system is that it is difficult to know the water availability in the entire rhizosphere because of the difficulty in obtaining a reliable *Ψ*_s_, mainly due to the temporal–spatial soil moisture heterogeneity and nocturnal transpiration (Kavanagh et al., 2007; Landsberg et al., 2017). In this study, we used *Populus euphratica*, an obligate phreatophyte species, to test the hypothesis. Because the root system of phreatophytes can reach and access the groundwater to avoid drought stress (Pockman and Sperry, 2000; Choat et al., 2012; Volaire, 2018), the Δ*Ψ* is largely determined by *Ψ*_l_ because *Ψ*_s_ is close to zero and the gravitational potential (−0.01MPa m^−1^) is ignorable for groundwater tables of a few meters (Scholander et al., 1965). *P. euphratica* mainly grows along the riverside of Tarim River at the north edge of the Taklimakan Desert, NW China. Previous greenhouse studies have found that stems of *P. euphratica* seedlings are highly vulnerable to cavitation (high *Ψ*_50_) and considerable *PLC* occurs at noon (Hukin et al., 2005), but there are no such field studies on this species. Thomas et al. (2008) and Gries et al. (2003) have found associations between *g*_s_, *VPD*, *k*_l_, and *Ψ*_l_, but the relationship between stomatal sensitivity to *VPD* and hydraulic traits is still poorly understood. We hypothesize that the stomatal response to *VPD* and xylem response to water potential are functionally converged to maintain a functional coherence and integrity of the hydraulic system of the tree.

## Materials and Methods

### Study Site

**Figure 1:**
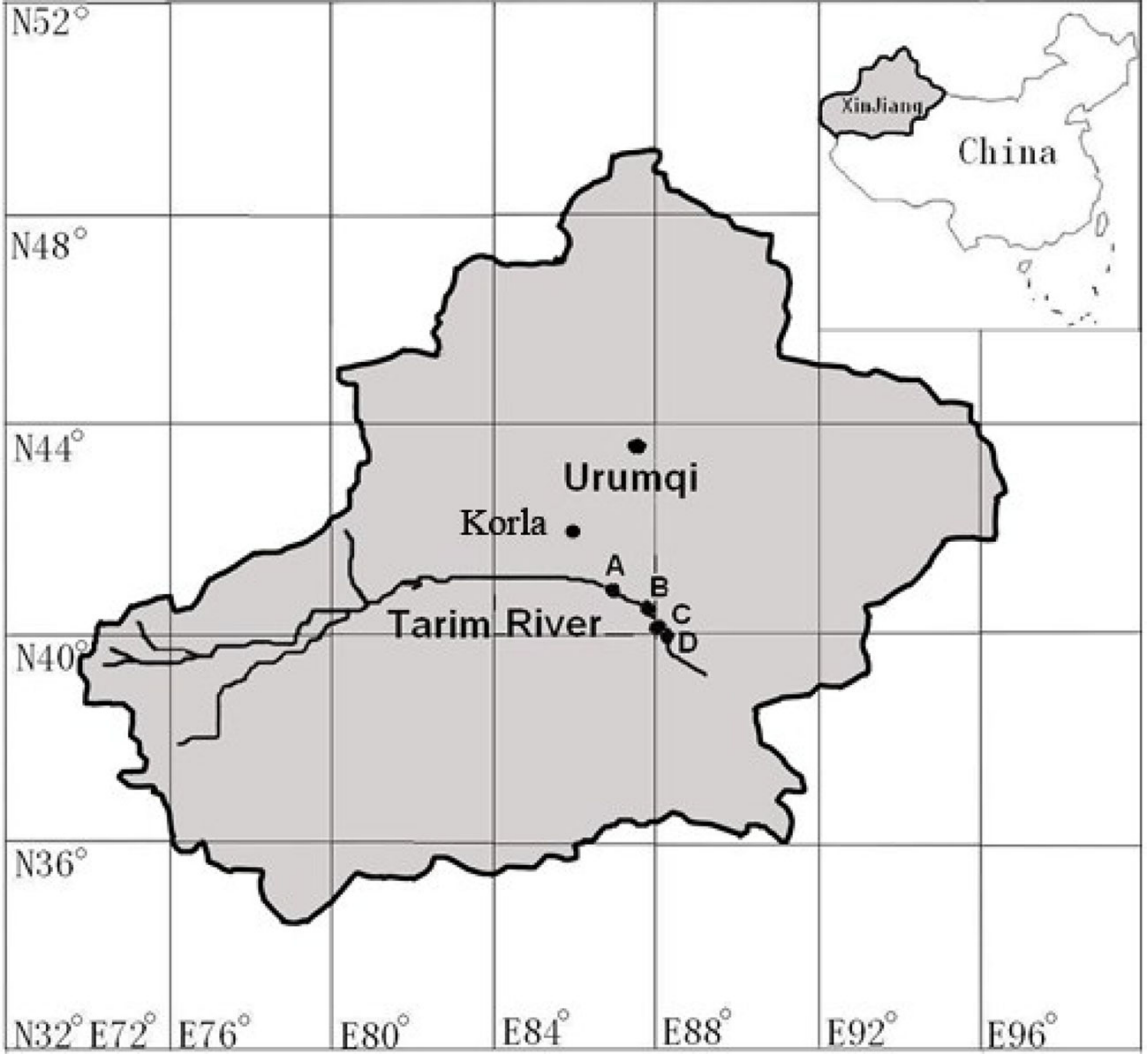
Geological locations of the four studied sites. Site name: A = 31 Tuan, B = 33 Tuan, C = Yingsu, D = Alagan.

The study was carried out at four sites along the downstream of Tarim River (elevation of 931 m) in the Xinjiang Uighur Autonomous Region, NW China (**Figure 1**). The four sites are located at least 138 km east of Korla, at the northern fringe of the Taklamakan desert. Korla has a warm temperate continental arid climate; the average length of the frost-free season is 210 days. The annual average temperature is about 11°C, the average annual precipitation is less than 60 mm, and the annual maximum evaporation is about 2,800 mm. The average maximum temperature and average minimum relative humidity in June are 30.9°C and 9.9% respectively.

The four sites had natural *P. euphratica* stands with relatively uniformly distributed trees. The stand density was 100–300 stems per hectare. Four to five trees with diameter at breast height of 30–40 cm were selected from each site and south-facing sunlit branchlets, and leaves at 1.3–1.5 m height from the outermost part of the lower canopy were selected for measuring *in situ g*_s_ and hydraulic characteristics. Two branches per tree were selected for the branch architecture measurement and one to two leaves per tree were used to measure *in situ g*_s_ response to *VPD*. The groundwater tables of the four sites measured at local wells within 1 km from the sites were 2.49, 3.49, 4.46, and 7.92 m, respectively, for site 31 Tuan (site A), 33 Tuan (site B), Yingsu (site C) and Alagan (site D).

### Measurements of *g*_s_ Response to *VPD*

*g*_s_ responses to *VPD* were measured on clear days in the field around noon (12:00 to 14:00 h) in late June under two sets of conditions: (1) controlled *VPD* and (2) un-controlled natural *VPD*, using a Li 6400 open gas exchange system (Li-Cor Cooperate, Lincoln, NE, USA). In the controlled-*VPD* measurements, a range of *VPD* from 0.8 to 3.5 kPa was achieved by using the apparatus on the equipment to vary the mixing ratio of water-vapor saturated air and dry air (after passing through desiccant). When the relative humidity in the leaf chamber exceeded 80% (*VPD* was about 0.8 kPa), the instrument displayed a warning sign of “High humidity alert”, *g*_s_ and intercellular carbon dioxide concentration (*C*_i_) readings fluctuated (*e.g.*, *C*_i_ fluctuated from negative to very large values), indicating the *g*_s_ measurement was not reliable. Therefore, data points with *VPD* values less than 1 kPa were discarded. Other environmental conditions in the leave cuvette were set as follows: Leaf temperature 31°C, Photosynthetically active radiation (PAR) 1,200 µmol m^−2^ s^−1^, CO_2_ concentration 390 µmol mol^−1^. Only steady-state *g*_s_ readings at each *VPD* were recorded (Dang et al., 1997; Dang, 2013). Measurements under un-controlled natural *VPD* were taken in June and again in July. The conditions in the leaf chamber were set the same in in the two measurements (Leaf temperature 31°C, PAR 1,200 µmol m^−2^ s^−1^, CO_2_ concentration 390 µmol mol^−1^). The −*m* and *b* were estimated using Equation 2 and the non-linear regression model with gnls () function of the R software (R Development Core, 2017).

### Leaf Water Potential and Branchlet Xylem Water Potential Measurements

The daily minimum leaf xylem water potential (*Ψ*_lmin_) was measured in the field between 12:00 and 14:00 using a Scholander pressure chamber (PMS Instrument, Corvallis, Oregon, USA). The measurements were taken on the same trees on which the *VPD* responses were measured. The daily minimum branchlet xylem water potential (*Ψ*_min_) was estimated according to the method of Pockman and Sperry (2000): a branchlet of similar size to that used in subsequent cavitation vulnerability measurements was selected and sealed in a plastic bag containing a moist paper towel for 30 min in darkness to allow the equilibration of water potential between leaves and the subtending branchlet before a leaf was sampled and the petiole water potential was measured.

### Cavitation Vulnerability Curve Measurement

A branchlet (50–70 cm long, 2–4 year-old) near that used for the *g*_s_–*VPD* response measurement was cut from each sample tree before sunrise (before 8:00 AM) to measure *k*_l_ and the cavitation vulnerability curve. The branchlet was wrapped in moist paper towels immediately after being cut and transported to the laboratory. The maximum vessel length was measured from six samples randomly chosen from all the four sites together, based on the method (pressurized gas bubble under water) of Jacobsen et al. (2007). Since the maximum measured vessel length was less than 21 cm, all the samples (7–10 per site) were re-cut to 22–24 cm under water, and all the measurements were carried out in an air-conditioned laboratory (26°C). The maximum flow rate was measured under 8 kPa hydrostatic pressure after air emboli were flushed out by perfusion with 110 kPa distilled water (flowing through 0.2 μm filter) for 30 min. Measurements were initiated after ~2 min when the flow rate stabilized. The weight of the collected efflux was measured every 30 s with a precision balance (Sartorius, BP221S, Göttingen, Germany) to obtain the flow rate. Maximum *k*_l_ was calculated by dividing the maximum flow rate by the total leaf area distal to the measured segment and by the pressure gradient. The leaf area was determined using a WinFOLIA system (Regent Instruments, Quebec City, Canada). *k*_s_ was calculated by dividing the maximum flow rate by the segment’s cross-section sapwood area. The total acropetal-end cross-section area of the branch segment was determined from its maximum and minimum diameter. The area of the pith was determined from its dimensions measured under a dissecting microscope equipped with a stage micrometer and subtracted from the above acropetal-end cross-section area to determine the cross-section sapwood area. The Huber value was calculated as the total cross-section sapwood area per unit leaf area (Tyree and Sperry, 1988).

The vulnerability of xylem to cavitation was characterized using a vulnerability curve which was measured using a Cavitation pressure chamber (PMS Instrument, Corvallis, Oregon, USA) according to Sperry and Saliendra (1994). A branch segment was inserted into a collar and sealed with both ends protruding. Air was injected into the collar at a set pressure, which was maintained for 15 min and then slowly decreased to 0.1 MPa. The hydraulic conductivity was then re-measured at a higher pressure. This procedure was repeated until at least 85% loss of hydraulic conductivity was reached. The *PLC* following each pressurization was calculated as *PLC* = 100 × (*K*_h_ − *K*_hi_)/*K*_h_, where *K*_hi_ is the hydraulic conductivity measured at pressure i. The vulnerability curve for each sample was fitted with an exponential sigmoidal equation (Pammenter and Vander Willigen, 1998):

(3) $$PLC=\frac{100}{1+e^{a\left(\Psi -\Psi_{50}\right)}}$$

where *Ψ* is the negative of the injection air pressure and coefficients *a* and *Ψ*_50_ are estimated using a non-linear regression model with gnls() function of R software (R Development Core, 2017). *Ψ*_50_ represents the xylem water potential at which 50% of the hydraulic conductance is lost, *a* represents the steepness of vulnerability curve. *k*_l_ at noon was estimated from *Ψ*_min_, the vulnerability curve, and maximum *k*_l_ (Pockman and Sperry, 2000; Johnson et al., 2009b).

### Wood Density and *k*_s_ Sensitivity to Water Potential

Wood density (*WD*) was measured on stem segments used in the measurement of vulnerability curves after the removal of pith and bark, and the fresh volume was measured by the Archimedes principle of water displacement. The dry mass was determined after drying at 104°C for 24 h. *WD* is expressed as dry mass per unit fresh volume (g cm^−3^).

Specific hydraulic conductance sensitivity to water potential [d(*k*_s_)/dln(−*Ψ*)] was calculated based on the method by Ewers et al. (2000): we related maximum *k*_s_, obtained at −*Ψ* = 0, to the branchlet *k*_s_ sensitivity to decreasing *Ψ* from −0.5 to −3.0 MPa. The slope of the −*Ψ*–*k*_s_ relationship was linearized by using the natural logarithm of –*Ψ*, and the logarithm transformation resulted in a good fit (R^2^ = 0.91 to 0.95).

### The Hydraulic Model

We used Equation 1 and the following equation:

(4) $$g_{l}=\left(g_{s}\cdot g_{bl}\right)/\left(g_{s}+g_{bl}\right)$$

to model the response of *g*_s_ to *VPD* at noon. We set constraints for *g*_sm_ (the maximum physiological *g*_s_ for *P. euphratica*), *g*_bl_, *K*_l_, and Δ*Ψ* according to the corresponding measured physiological range for *P. euphratica*. It is assumed that *g*_s_ had an upper limit of *g*_sm_ which was set as 1,000 mmol m^−2^ s^−1^, based on our field measurements and the reported values for poplars (Hacke, 2015). The *g*_bl_ for desert environment was set as 2,000 mmol m^−2^ s^−1^ (Comstock and Mencuccini, 1998). *K*_l_ at noon was set between 0.5 and 4.0 mmol MPa^−1^ m^−2^ s^−1^. Δ*Ψ* at noon (*Ψ*_s_ − *Ψ*_lmin_) was set at 2.0 to 3.2 MPa according to the measured range of *Ψ*_lmin_ in the field. *VPD* was allowed to vary between 1 and 4 kPa, similar to the range observed in the field (Gries et al., 2003). Before running the simulation, we calculated *K*_l_ at noon from the estimated *k*_l_ at noon, assuming hydraulic path length = underground water table + sample height + branch length, and hydraulic conductance was uniformly distributed along the flow path from soil to branchlet (Pockman and Sperry, 2000). Note the unit of *k*_l_ is kg m^−1^ MPa^−1^ s^−1^, and the unit of *K*_l_ is mmol m^−2^ MPa^−1^ s^−1^. We converted the unit *VPD* into the unit of mol mol^−1^ as required by the model. We relaxed the assumption of constant Δ*Ψ* when exploring the relationship between *K*_l_ and −*m/b*. By allowing *K*_l_ and Δ*Ψ* to vary simultaneously, the *g*_s_ responses to *VPD* (−*m/b*) under all the combinations of *K*_l_ and Δ*Ψ* were solved. The simulation procedure was as follows (Oren et al., 1999): 1) the *K*_l_ and Δ*Ψ* were assigned to specific values; 2) *g*_l_ as the function of *VPD* was calculated from Equation 1; 3) *g*_s_ was solved from Equation 4; 4) when *g*_s_ > *g*_sm_ (occurred occasionally), we set *g*_s_ = *g*_sm_ and re-solved the equation for Δ*Ψ*; 5) the calculated *g*_s_ and assigned *VPD* were fitted by a non-linear regression model with the gnls () function of R software (R Development Core, 2017) to obtain −*m/b* from Equation 2; 6) a semi-contour plot −*m/b versus* Δ*Ψ* and *K*_l_ was constructed.

### Statistical Analyses

The distribution normality of branchlet xylem hydraulic traits and leaf water status data were tested by calculating the Shapiro–Wilk W statistics for each sample (n = 4–5 for leaf water status variables, n = 7–10 for branchlet xylem hydraulic traits). Differences between sites were tested using Kruskl–Wallis H test if P(W) < 0.05. The Spearman rank correlation analysis was applied to investigate correlations among branchlet xylem hydraulic traits. ANOVA and Pearson correlation analysis were subsequently conducted if P(W) > 0.05. We conducted all the statistical analyses using R version 3.4.0 (R Development Core, 2017).

## Results

*g*_s_, *Ψ*_lmin_, *b* were significantly lower, and *VPD* was significant higher at Alagan than other sites (**Table 2**). Transpiration rate (*T*_r_), −*m*, and −*m*/*b* (0.57–0.61) were not significantly different among the four sites (**Table 2**). Differences in all the traits were smallest between 31 Tuan site and Yingsu site than among all the sites.

**Table 2:** Means and standard error of the mean for *g*_s_, *T*_r_, *VPD*, *Ψ*_lmin_, *−m*, *b* and *−m/b* at the four sites.

Population	*g*_s_	*T*_r_	*VPD*	*Ψ*_lmin_	*−m*	*b*	*−m/b*
31 Tuan	536 ± 68^a^	7.28 ± 1.48^a^	1.56 ± 0.10^b^	*−*2.30 ± 0.03^a^	518.9 ± 130.6^a^	802.5 ± 102.9^a^	0.61 ± 0.10^a^
33 Tuan	396 ± 39^ab^	7.62 ± 0.63^a^	1.96 ± 0.10^b^	*−*2.55 ± 0.05^a^	333.7 ± 54.8^a^	588.1 ± 72.0^ab^	0.57 ± 0.07^a^
Yingsu	490 ± 41^a^	8.73 ± 0.90^a^	1.88 ± 0.22^b^	*−*2.45 ± 0.06^a^	435.2 ± 60.0^a^	769.7 ± 8.3^a^	0.57 ± 0.08^a^
Alagan	328 ± 39^b^	8.07 ± 0.65^a^	2.46 ± 0.08^a^	*−*3.01 ± 0.09^b^	328.3 ± 33.2^a^	577.1 ± 35.7^b^	0.58 ± 0.08^a^
P value	≤0.05	>0.05	≤0.05	≤0.05	>0.05	>0.05	>0.05

Symbols, their definition and units are provided in **Table 1**.

Different letters among populations indicate significant difference at p = 0.05.

The branchlet hydraulic architecture of *P. euphratica* also varied with site (**Table 3**). Yingsu had significantly greater *k*_s_, *k*_l_, and *d(k*_s_*)/dln(−Ψ)* than the other three sites (**Table 3**). Huber value was lowest at Alagan and highest at 33 Tuan among the four sites although their differences from 31 Tuan and Yingsu were not statistically significant (**Table 3**). *WD* was lowest at 31 Tuan and highest at Alagan, but their differences from 33 Tuan and Yingsu were not statistically significant (**Table 3**). *Ψ*_min_ had the same trend as *Ψ*_lmin_, *i.e.*, significantly more negative at Alagan than at all other sites, while there was no significant difference among the other sites (**Tables 2** and **3**). *Ψ*_50_ was most negative at Alagan (−2.22 MPa) and the least negative at Yingsu (−1.22 MPa) among the four sites, but the trend for *a* was the opposite (**Table 3**).

**Table 3:** Means and the standard error of the mean for *k*_s_, *k*_l_, *WD*, *Ψ*_min_, Huber value, *Ψ*_50_, d(*k*_s_)/dln(*−Ψ*), and *a* at the four sites.

Population	*k*_s_	*k*_l_(×10^−4^)	*WD*	*Ψ*_min_	*Huber*(×10^−4^)	*Ψ*_50_	*a*	*d(k*_s_*)/dln(*^−^*Ψ)*
31 Tuan	1.52 ± 0.16^b^	3.51 ± 0.43^b^	0.405 ± 0.035^b^	*−*2.14 ± 0.06^a^	2.79 ± 0.34^ab^	*−*1.39 ± 0.12^ab^	1.71 ± 0.16^a^	0.59 ± 0.06^b^
33 Tuan	1.51 ± 0.34^b^	3.68 ± 0.28^b^	0.491 ± 0.007^ab^	*−*2.27 ± 0.16^a^	3.05 ± 0.35^a^	*−*1.67 ± 0.15^b^	1.35 ± 0.15^ab^	0.49 ± 0.15^b^
Yingsu	2.94 ± 0.36^a^	6.63 ± 0.84^a^	0.474 ± 0.006^ab^	*−*2.23 ± 0.08^a^	2.40 ± 0.36^ab^	*−*1.22 ± 0.23^a^	1.72 ± 0.19^a^	1.08 ± 0.20^a^
Alagan	2.02 ± 0.49^b^	3.04 ± 0.35^b^	0.496 ± 0.010^a^	*−*2.73 ± 0.03^b^	31.63 ± 0.22^b^	*−*2.22 ± 0.11^c^	0.94 ± 0.08^b^	0.83 ± 0.17^ab^
P value	≤0.01	≤0.001	≤0.01	≤0.001	≤0.01	≤0.001	≤0.01	≤0.05

Symbols, their definition, and units are given in **Table 1**.

Different letters among populations indicate significant difference at p = 0.05.

**Figure 2:**
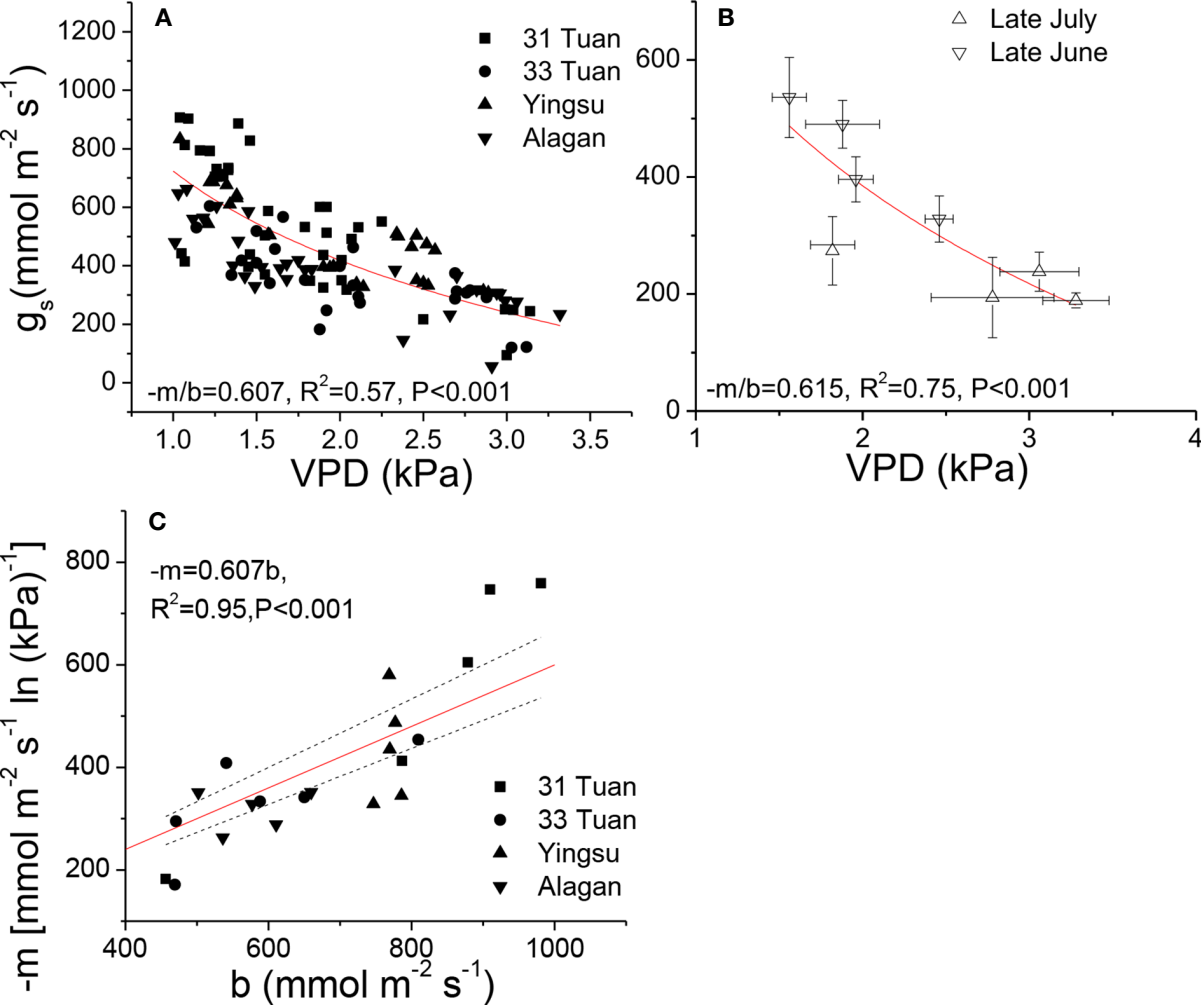
Relationships between *g*_s_ and *VPD* **(A, B)**, and the relationship between −*m* and *b* **(C)** of *Populus euphratica* among the four sites. The measurements were taken by manipulating *VPD* in late June **(A, C)**, and natural *VPD* in late June and late July **(B)**. The curves were generated by using the hydraulic model (Equation 1). In **(C)**, red line represents the theoretical line of the expected relationship (*−m* = 0.6*b*), black dash lines represent 95% confidence intervals of linear regression between observed *−m* and *b*.

As there was no significant difference in *−m/b* among the sites, we pooled all the *VPD* response data from the four sites and evaluated the general relationship between *g*_s_ and *VPD* for the species. We fit Equation 1 separately for the two sets of *VPD* response data. The estimated −*m/b* was 0.607 for the controlled-*VPD* data set and 0.615 for the natural *VPD* field measurement (**Figures 2A, B****)**. −*m* was positively correlated with *b* across the individuals of the four sites (**Figure 2C**).

**Figure 3:**
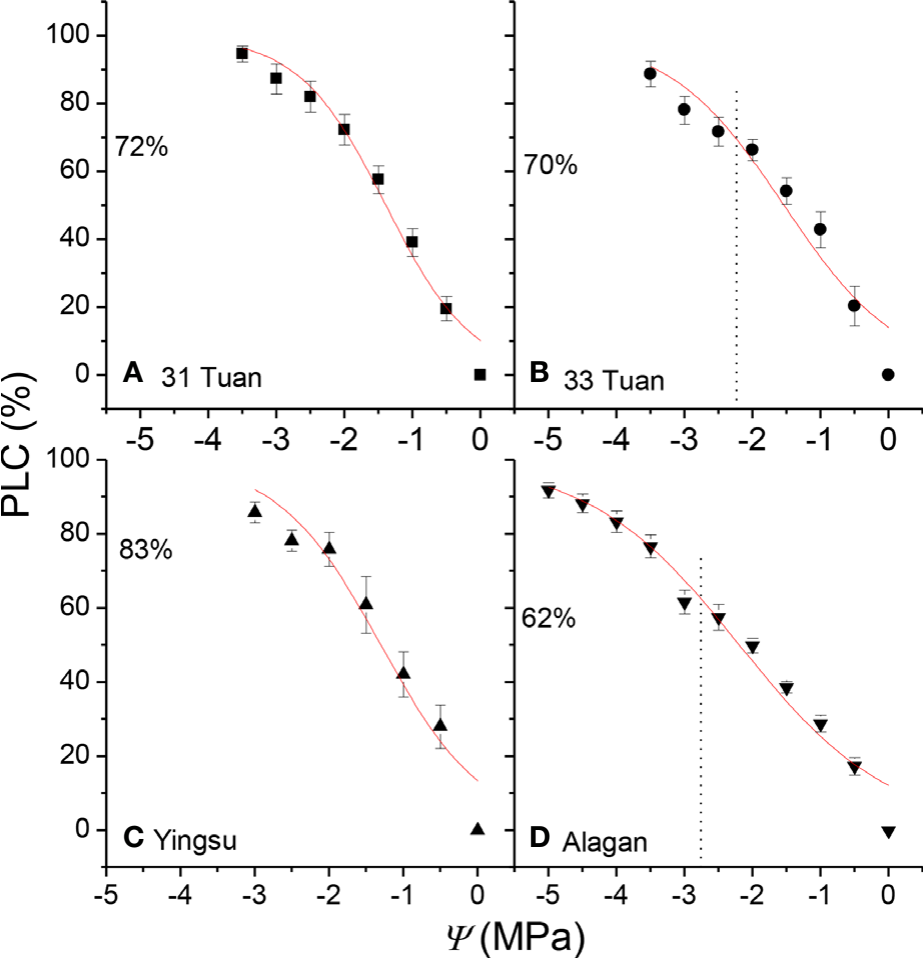
*PLC* as a function of *Ψ* for the four populations of *Populus euphratica* (means ± 1 se). The curves were fitted using all data points. The vertical dotted line indicates the position of measured *Ψ*_min_; the horizontal dotted line indicates the percentage loss of hydraulic conductivity at *Ψ*_min_. The number under the horizontal dotted line is the *PLC* at noon. See **Table 1** for the definition of symbols.

The estimated *PLC* ranged from 62% at Alagan site to 83% at Yingsu site (**Figure 3**). The corresponding estimated *k*_l_ at noon for 31 Tuan, 33 Tuan, Yingsu, and Alagan sites was 1.11 × 10^−4^, 1.09 × 10^−4^, 1.12 × 10^−4^, and 1.16 × 10^−4^ kg m^−1^ MPa^−1^ s^−1^, respectively.

**Figure 4:**
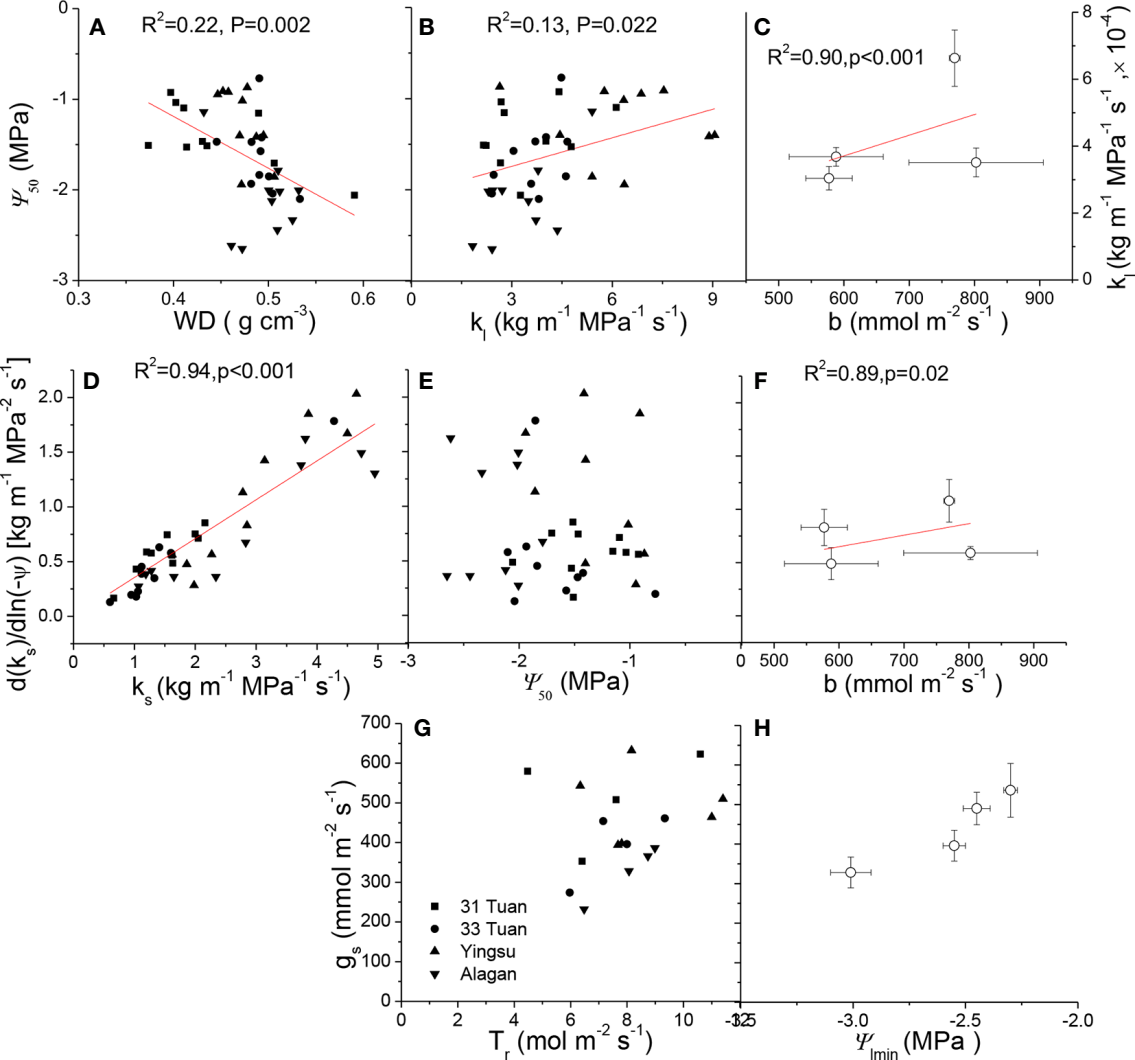
*Ψ*_50_ as a function of: **(A)** *WD* and **(B)** *k*_l._ *k*_l_ as a function of *b* at the population scale **(C)**. d(*k*_s_)/dln(−*Ψ*) as a function of: **(D)** *k*_s_, **(E)** *Ψ*_50_, and **(F)** *b* at the population scale of *Populus euphratica* at the four sites. *g*_s_ as a function of: **(G)** *T*_r_, and **(H)** *Ψ*_min_ at the population scale of *Populus euphratica* at the four sites. In **Figures 4C, F** and **H** , data were shown as mean ± 1 se. For relationships at the population scale, the four points are the means of the four sites.

**Figure 5:**
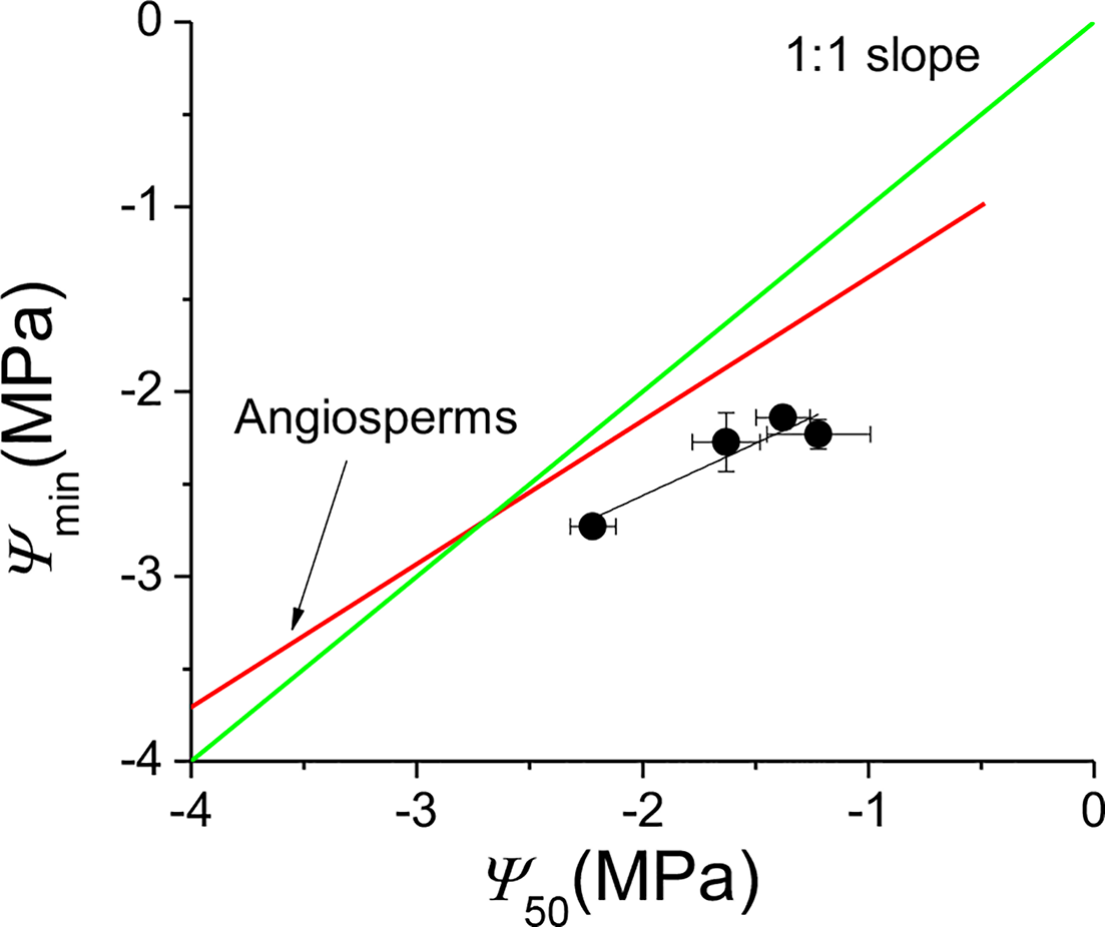
*Ψ*_min_ as a function of *Ψ*_50_ for *Populus euphratica* at the four sites. The four black points are the means of the four sites. The red line is the regression line fitted using measurements for angiosperm species in the world (Choat et al., 2012). The F value and probability for the regression black line of *Populus euphratica* at the four sites are 14.76 and 0.061, respectively. The green line is the 1:1 regression line between *Ψ*_min_ and *Ψ*_50_. Errors bars represent ± se.

*k*_s_ was positively correlated with *k*_l_ (F = 6.637, P = 0.014), negatively correlated with Huber value (F = 19.407, P < 0.001) (**Table 4**). However, *k*_s_ showed no significant relationship with safety (*Ψ*_50_) (F = 1.917, P = 0.175) (**Table 4**). There was a negative association between *Ψ*_50_ and *WD* (F = 8.566, P = 0.006) across the four sites (**Table 4**, **Figure 4A**). There was a positive correlation between *Ψ*_50_ and *k*_l_ (F = 5.937, P = 0.017) (**Table 4**, **Figure 4B**); *k*_l_ also showed a positive correlation with the reference stomatal conductance at 1.0 kPa (F = 37.274, P = 0.009) at the population scale (**Figure 4C**). d(*k*_s_)/dln(−*Ψ*) was positively correlated with *k*_s_ (F = 680.782, P < 0.001) (**Figure 4D**) across the individuals, and *b* at the population scale (F = 33.249, P = 0.01) (**Figure 4F**), but had no relationship with *Ψ*_50_ (F = 0.438, P = 0.51) (**Figure 4E**). There was no association between *g*_s_ and *Ψ*_lmin_ at the population scale (F = 12.047, P = 0.071) (**Figure 4H**). There was no significant relationship between *g*_s_ and *T*_r_ (F = 0.99, P = 0.33) (**Figure 4G**). There was also a marginally positive association (F = 13.048, P = 0.061) between *Ψ*_50_ and *Ψ*_min_ across the four sites, and the slope of this relationship is similar to the slope for other angiosperm species in the world (**Figure 5**). The safety margin (*Ψ*_min_ − *Ψ*_50_) was negative (*i.e.*, below the 1:1 line in **Figure 5**) because the actual measured *Ψ*_min_ was more negative than *Ψ*_50_ and the actual PLC was greater than 50%. *g*_s_ increased with increasing (*i.e.*, becoming less negative) *Ψ*_min_, and the rate of increase was greater at sites with less negative *Ψ*_min_ (**Figure 4H**).

**Table 4:** Pearson correlations between branchlet hydraulic traits of *Populus euphratica*.

	*k_s_*	*k_l_*	*WD*	*Huber*	*Ψ*_50_
*k_s_*	1				
*k_l_*	0.399^**^	1			
*WD*	0.236^ns^	0.06 ^ns^	1		
*Huber*	^−^0.581^***^	0.265 ^ns^	^−^0.172 ^ns^	1	
*Ψ*_50_	^−^0.228 ^ns^	0.355^*^	^−^0.443^**^	0.387^**^	1

Symbols, their definition, and units can be found in **Table 1**.

*, **, *** and ns denote significance at P ≤ 0.05, P ≤ 0.01, P ≤ 0.001 and no significant difference, respectively.

**Figure 6:**
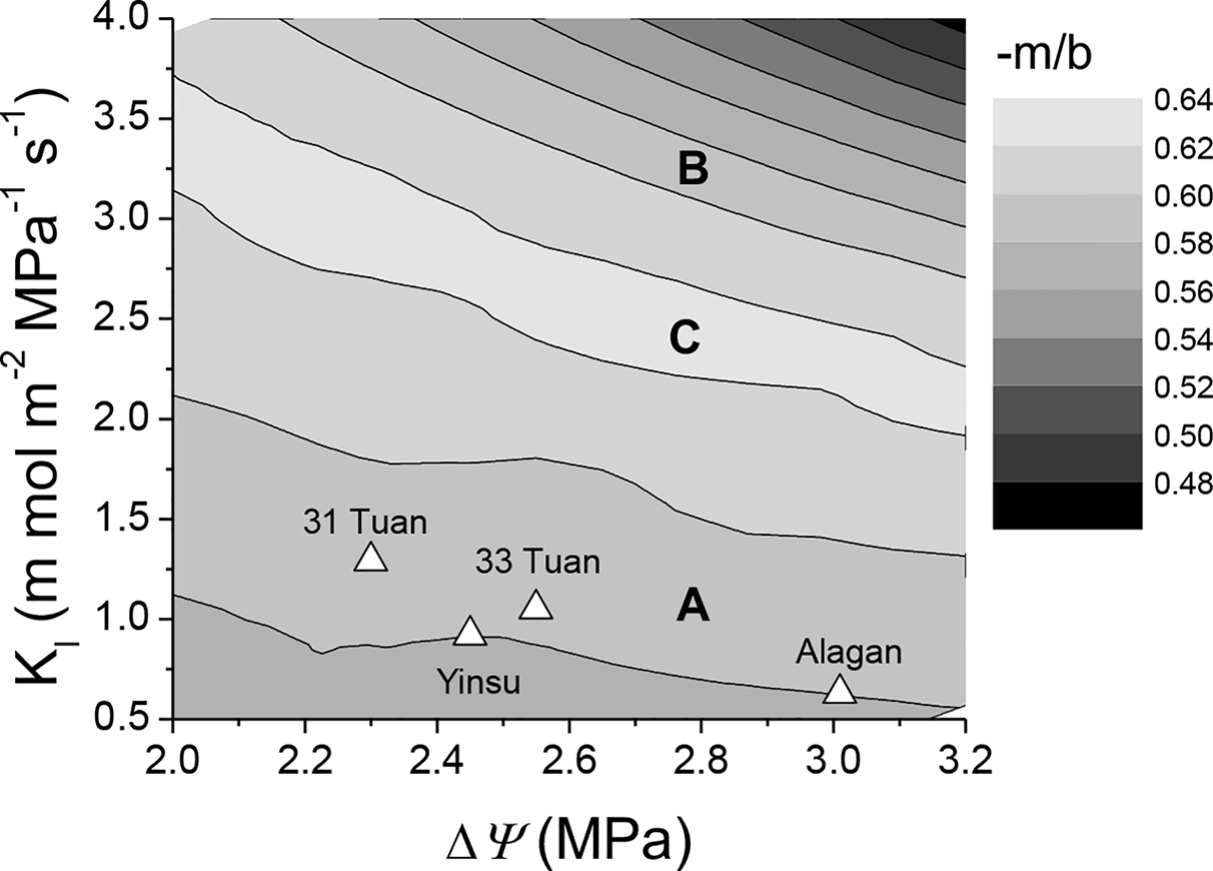
Semi-contour plots of *−m/b* as dependent on Δ*Ψ* and *K*_l_. Plots are derived from the hydraulic model (Equation 1). The simulated *−m/b* at the four studied sites are shown as (Δ). *A* and *B* in the figure represent belts with same *−m/b* but different *K*_l_ at noon, *C* represents belt with higher *−m/b* than *A* and *B*, but its *K*_l_ at noon is between those of *A* and *B*. Note the unit of *K*_l_ is mmol m^−2^ MPa^−1^ s^−1^.

**Figure 6** demonstrated the effect of −*m*/*b* on the relationship between *K*_l_ and Δ*Ψ*. The average values of *K*_l_ and Δ*Ψ* around noon for the four sites all fell well within the range of 0.58–0.60 −*m*/*b*. Belt A and belt B in **Figure 6** were within the same −*m/b* range (0.58–0.60) but different *K*_l_ at noon. Belt C had a −*m/b* range of 0.62–0.64, but its *K*_l_ at noon was higher than belt A but lower than belt B.

## Discussion

The *Ψ*_50_ values measured in this study are within the range of values reported for poplar trees around the world, *e.g.*, −1.30 MPa for *P. tremula*, −2.95 MPa for *P. nigra* (Choat et al., 2012), −0.69 MPa for *P. deltoids*, and −2.75 MPa for *P. tremuloides* (Fichot et al., 2015). Hukin et al. (2005) have reported that *P. euphratica* seedlings have a *Ψ*_50_ for xylem cavitation of only −0.7 MPa, while the field-grown *P. euphratica* trees in our study had an average *Ψ*_50_ of −1.63 MPa across the four sites. These results suggest that the seedlings of *P. euphratica* may be much more vulnerable to xylem cavitation than trees at a later developmental stage. Although the seedlings of *P. euphratica* were measured at a different location in a different study (Hukin et al., 2005) from the larger trees, the response trend is consistent with that of another poplar species, *P. tremuloides*, the field grown trees which have a *Ψ*_50_ of −2.75 MPa (Sperry and Sullivan, 1992) while its seedlings only −0.68 to −0.84 MPa (Way et al., 2013). Furthermore, the value of *Ψ*_50_ that we measured on *P. euphratica* in this study (−1.63 MPa) is much less negative than the reported values for other drought resistant tree species, *e.g.*, −2.75 MPa for *P. tremuloides* (Sperry and Sullivan, 1992), −8.42 MPa for *Pistacia terebinthus* (Maherali et al., 2004), suggesting that *P. euphratica* may be more vulnerable to cavitation than other drought tolerant desert species. Our measurement of *PLC* was in line with the mid-day *PLC* of 76% reported by Zhou et al. (2013).

The results of this study suggest that *P. euphratica* is a mesic-adapted species. In a mesic-adapted species, *g*_s_ generally has a much tighter relationship with *VPD* (**Figures 2A, C****)** than with *k*_s_ (F = 0.02, P = 0.89 at the population scale) or *T*_r_ (**Figure 4G**) (Thomas et al., 2008; Yu et al., 2019), and −*m* is generally converged at 0.6 when standardized by *b* (Oren et al., 1999). The −*m/b* value of 0.6 is consistent with the prediction of the hydraulic model (Equation 1) that assumes that stomatal conductance controls the leaf water potential and transpiration rate under full irradiance. Both our controlled-*VPD* experiment and the measurements of *g*_s_ response to natural *VPD* measured under field conditions across all four sites confirmed that the −*m/b* value was approximately 0.6 for *P. euphratica*. The results also support the hypothesis that the hydraulic homeostasis of phreatophyte species is restricted by transpiration water demand (*VPD*) but not by the water supply in the soil because their root system can uptake water directly from the groundwater, and thus their water supply remains largely stable throughout the year (Thomas et al., 2000; Bruelheide et al., 2003). The nearly isohydric behavior could also be demonstrated by the close association between −*m* and *b* across the individuals from the four populations (**Figure 2C**). The high xylem vulnerability and the pattern of *g*_s_ response to *VPD* found this study provide physiological explanations for some of the ecological phenomena that are often observed in the field, *e.g.*, very little sexual reproduction (Hukin et al., 2005), extensive clonal growth (Bruelheide and Jandt, 2004). We observed that there were very few seedlings at the four sites of this study. The limited number of seedlings on the sites may have been a result of the inability of seedlings to access the ground water, particularly during the dry season. The groundwater table generally deepens as a result of the management of the river system (Chen et al., 2015). In order to survive a drought spell, the root system of the seedlings must be able to reach and access the groundwater because of the high vulnerability to xylem cavitation and the inability of the species to effectively control water loss when the transpirational demand is high (*i.e.*, mesic *g*_s_ response to *VPD*). Because the root system of seedlings generally cannot penetrate deep enough to tap the groundwater as the ground table deepens or if they grow far away from the river bank, the mortality rate of tree seedlings is very high (Chen et al., 2015), leading to a lower rate of successful sexual regeneration. Consequently, the proportion of vegetative regeneration from suckering generally increases with increasing distance from the river bank. Similar phenomena on *P. euphratica* have been reported by other studies (*e.g.*, Wu et al., 2010). These results suggest that the distribution of this species in the desert primarily depends on its access to groundwater (Gries et al., 2003).

The “safety margin” (*Ψ*_min_ − *Ψ*_50_) of *P. euphratica* ranged from −0.5 to −1.01 MPa across the four sites in this study. While these values are within the general range of values reported for other tree species in the world that grow under comparable environmental conditions to those of our study sites (Choat et al., 2012), *P. euphratica* tended to have a more negative *Ψ*_min_ than other species with the same *Ψ*_50_ (**Figure 5**). The linear regression line between *Ψ*_min_ and *Ψ*_50_ (marginally significant) had a similar slope to that of angiosperm species in the literature (Choat et al., 2012). suggesting that the species tended to regulate stomatal aperture to maintain water homeostasis but was less able to do so. However, the negative value of the safety margin suggests that *P. euphratica* operated beyond the hydraulic safety margin and thus suffered more than 50% loss of hydraulic conductivity around noon. Indeed, the *PLC* at noon ranged from 62% at Alagan to 83% at Yingsu site. Since it is generally believed that plants can fine-tune *PLC* to avoid catastrophic hydraulic dysfunction (Tyree and Sperry, 1988; Macinnis-Ng et al., 2004), it is puzzling why such large differences in *PLC* occurred in *P. euphratica* at different sites. We thus proposed that plants can fine-tune *K*_l_ *via* precise control over *PLC* to maintain a constant −*m*/*b*, according to the prediction of Equation 1 and Equation 2 (Oren et al., 1999; Ewers et al., 2000), which can provide insight into the mechanism underpinning the large variation of *PLC* in *P. euphratica*. The results further suggest that *P. euphratica* had a greater ability to restore cavitated xylem vessels daily than most angiosperm species. However, the factor or factors responsible for such a high ability are not clear and warrant investigations.

The results of this study suggest that the hydraulic model (Equation 1) and/or its assumptions may need to be modified when used to examine the relationship between *K*_l_ and −*m/b*. The model predicts that if *K*_l_ decreases due to xylem cavitation, the −*m/b* will increase because a greater stomatal response is required to keep transpiration and Δ*Ψ* (Soil water potential minus leaf water potential) constant (Oren et al., 1999; Landsberg et al., 2017). However, the −*m/b* in the current study was relatively constant (close to 0.6) across the four sites despite the large declines in *K*_l_ at noon. Furthermore, the model assumes that Δ*Ψ* remains constant when *K*_l_ varies, which unlikely occurs in nature. In this study, we allowed *K*_l_ and Δ*Ψ* to vary concurrently to relax the assumptions and set values for *K*_l_, Δ*Ψ*, *g*_sm_, and *g*_bl_ based on the measured physiological ranges for *P. euphratica* in our investigation of the relationship between *K*_l_ and −*m/b*.

The output of the hydraulic model with our modifications was supported by our field measurements. The modeled relationship between *K*_l_ and Δ*Ψ* at noon for our four sites was within the band of 0.58–0.60 −*m/b* (**Figure 6**), and the −*m/b* range calculated from our field measurements was 0.57–0.61. The *in situ* native midday *PLC* for *P. euphratica* (76%) measured by Zhou et al. (2013) along the Arim River is also consistent with our modeled value. A 1:1 correspondence between native embolism and the embolism predicted from vulnerability curves for desert plants is also reported by Pockman and Sperry (2000). These results suggest that correctly constructed VC curves (Wheeler et al., 2013) and hydraulic models can reliably predict native embolism in the field. It is also reported that xylem embolism is likely a critical element for the decrease of leaf hydraulic conductance during the daytime (Kikuta et al., 1997; Woodruff et al., 2007; Johnson et al., 2009a; Zhang et al., 2016), particularly for poplar species (Laur and Hacke, 2014; Scoffoni et al., 2017). *P. euphratica* could tolerate more than 50% xylem cavitation around noon and that it regulated *PLC*s based on the different conditions of the four sites in order to achieve a similar stomatal sensitivity under the condition where water supply and irradiance were not limited. This hydraulic strategy might be critical for *P. euphratica* to maximize its carbon gain and facilitate its growth in an arid environment. It can be inferred that *P. euphratica* trees may have the capacity of refilling the embolized xylem vessels or producing new vessels to restore the hydraulic capacity quickly (Gleason et al., 2016; Choat et al., 2018). However, the literature indicates that not all the species have the capacity to refill caviated vessels (Charrier et al., 2016; Torres-Ruiz, 2020). Further research in this area is warranted.

There are generally considerable variations in *PLC* at mid-day minimum xylem water potential among and within species (Pockman and Sperry, 2000; Johnson et al., 2009b; Fan et al., 2018), and based on the *PLC* at the mid-day water potential, a species can be classified into one of the two strategic groups: the conservative group (*PLC* < 50%) or the radical group (50% <*PLC* < 100%). Interestingly, the results of model simulation in the present study indicate that the two strategy groups could have similar stomatal sensitivity (*e.g.*, belt A for the radical strategy and belt B for the conservative strategy in **Figure 6** had the same range of stomatal sensitivity), suggesting that maintaining the theoretical threshold stomatal sensitivity (−*m/b* = 0.6) is probably critical for the fitness of mesic species regardless of which strategy they adopt. A *−m/b* above 0.62 (Belt C in **Figure 6**, with smaller *PLC* than the radical strategy and greater *PLC* than the conservative strategy) might be detrimental to photosynthesis because a small increase in *VPD* would induce a large decline in stomatal conductance and thus CO_2_ supply for photosynthesis even when water supply and irradiance are not limited, possibly reducing the competitiveness of the species. However, the reason why *P. euphratica* has adopted the radical strategy (belt A in **Figure 6**) instead of the conservative strategy (belt B in **Figure 6**) remains unknown. It is possible that xylem cavitation may help plants to survive drought stress by rationing water use (Sperry, 1995) and temporally releasing the effects of water stress for a portion of the tree (Hölttä et al., 2011). It is also worth noting that *P. fremontii* (a riparian species in Sonoran desert), a comparable species to *P. euphratica*, has 16.5–31.97% embolism at noon (Pockman and Sperry, 2000) and is likely located in belt B of **Figure 6** and has adopted a conservative strategy.

The result that increased cavitation resistance was linked to increased wood density (**Figure 4A**) is expected because denser wood tends to be better able to sustain the compressive forces generated by lower negative pressures and to minimize air permeability that might cause xylem cavitation (Pockman and Sperry, 2000; Hacke et al., 2001; Bucci et al., 2013). Further, the positive relationship between *k*_l_ and *Ψ*_50_ (**Figure 4B**) suggests there was a functional trade-off between efficiency and safety at the leaf level. The positive relationship between k_s_ and d(*k*_s_)/dln(−*Ψ*) (**Figure 4E**) is coherent with the positive relationships between stomatal sensitivity to *VPD* and stomatal conductance at low *VPD* (*i.e.*, 1.0 kPa) and between leaf hydraulic conductance and stomatal conductance at low *VPD*, providing evidence to support the functional convergence between xylem and leaves. Furthermore, faster growing species or populations tend to have lower wood density, higher stomatal and xylem conductance but lower drought resistance. The curvilinear relationship between *g*_s_ and *Ψ*_min_ suggests that trees with less negative *Ψ*_min_ had higher but more sensitive *g*_s_, which also coincided with lower wood density and higher hydraulic conductance as well as sites with shallower water tables. The results of this study provide physiological evidence for the mechanisms governing the tradeoff between growth rate, anatomy, physiological functioning, and stress resistance.

Transpiration is controlled by both vapor pressure deficit and leaf conductance. Therefore, it is not surprising that *g*_s_ was not significantly correlated to *T*_r_ (**Figure 4G**). The leaf conductance in turn is controlled by *VPD* and the internal water status as demonstrated by Equations 1 and 2. Equation 1 demonstrates that leaf conductance is the linkage between the internal water relations in the tree and the moisture conditions of the ambient air. This conclusion can further enforced the coherent functional relationships discussed in the previous paragraph, such as the significant, linear relationship between d(*k*_s_)/dln(−*Ψ*) and *k*_s_ (**Figure 4D**). This relationship could facilitate the fine-tuning of *PLC* to sustain transpiration (Ewers et al., 2000) across the individuals (**Table 2**, **Figure 2**). More importantly, the lack of significant relationship between *g*_s_ and *T*r/*Ψ*_lmin_ (**Figures 4G, H****)** indicates that the hydraulic behavior of *P. euphratica* resembled that of an isohydric species, which is in consistence with the observation on *P. euramericana* (Tardieu and Simonneau, 1998) and several poplar genotypes (Navarro et al., 2018). The nearly isohydric behavior also indicates higher *b* (same meaning as −*m*, Oren et al., 1999) is functionally associated with higher d(*k*_s_)/dln(−*Ψ*) and higher *k*_l_ (at the population scale) (**Figures 4C, F****)**, consistent with the observation that stomata respond to changes in branchlet hydraulic conductance in a manner of feedback response to leaf water status (Saliendra et al., 1995). The results can be explained solely by hydraulic signaling or by an interaction between hydraulic and chemical signaling in the control of stomatal conductance (Tardieu and Simonneau, 1998; Comstock, 2002; Buckley, 2019; Qu et al., 2019).

In summary, this study demonstrates that the hydraulic architecture of branchlets and stomatal response to *VPD* were well coordinated with each other so that the water homeostasis of *P. euphratica* was maintained in the desert environment. The high xylem vulnerability to cavitation and the pattern of *g*_s_ response to *VPD* measured in the field further corroborated previous conclusions that the distribution and growth of *P. euphratica* in the desert solely depend on its access to groundwater (Gries et al., 2003; Hukin et al., 2005; Thomas et al., 2008). Thus, the populations of this phreatophyte species may decline if and when the groundwater table deepens as a result of reduced precipitation induced by global climate change, river management, or dam constructions (Zhou, 1993; Gries et al., 2003; Chen et al., 2015). We also demonstrated that the observed *−m/b* of *P. euphratica* is consistent with the theoretical value derived from a simple hydraulic model when the assumption of constant Δ*Ψ* was relaxed. Our results demonstrate that model simulations can potentially explain the wide range of variations in *PLC* across and within woody species that is often observed in the field but further research efforts in this area is warranted.

## Data Availability Statement

The raw data supporting the conclusions of this article will be made available by the authors, without undue reservation.

## Author Contributions

D-YF and S-RZ designed the experiment. D-YF, C-DJ, X-WX, and X-FY carried out the experiment. D-YF, S-RZ, C-YX, and Q-LD performed the statistical analyses and drafted the manuscript. W-FZ assisted in the experiment. All authors commented on the manuscript. All authors contributed to the article and approved the submitted version.

## Funding

This study was financially supported by the National Key Research and Development Program of China (2016YFA0600802), State Key Project of Research and Development Plan (2016YFC0502104) and the Science and Technology Project of Beijing (Z171100004417019).

## Conflict of Interest

The authors declare that the research was conducted in the absence of any commercial or financial relationships that could be construed as a potential conflict of interest.
